# DMSO Represses Inflammatory Cytokine Production from Human Blood Cells and Reduces Autoimmune Arthritis

**DOI:** 10.1371/journal.pone.0152538

**Published:** 2016-03-31

**Authors:** Ingrid Elisia, Hisae Nakamura, Vivian Lam, Elyse Hofs, Rachel Cederberg, Jessica Cait, Michael R. Hughes, Leora Lee, William Jia, Hans H. Adomat, Emma S. Guns, Kelly M. McNagny, Ismael Samudio, Gerald Krystal

**Affiliations:** 1 The Terry Fox Laboratory, British Columbia Cancer Agency, Vancouver, B.C., Canada; 2 The Biomedical Research Centre, University of British Columbia, Vancouver, B.C., Canada; 3 The Brain Research Centre, University of British Columbia, Vancouver, B.C., Canada; 4 The Vancouver Prostate Centre at Vancouver General Hospital, Vancouver, B.C., Canada; University of Leuven, Rega Institute, BELGIUM

## Abstract

Dimethyl sulfoxide (DMSO) is currently used as an alternative treatment for various inflammatory conditions as well as for cancer. Despite its widespread use, there is a paucity of data regarding its safety and efficacy as well as its mechanism of action in human cells. Herein, we demonstrate that DMSO has *ex-vivo* anti-inflammatory activity using *Escherichia coli*- (*E*. *coli*) and herpes simplex virus-1 (HSV-1)-stimulated whole human blood. Specifically, we found that between 0.5%– 2%, DMSO significantly suppressed the expression of many pro-inflammatory cytokines/chemokines and prostaglandin E_2_ (PGE_2_). However, a significant reduction in monocyte viability was also observed at 2% DMSO, suggesting a narrow window of efficacy. Anti-inflammatory concentrations of DMSO suppressed *E*. *coli*-induced ERK1/2, p38, JNK and Akt phosphorylation, suggesting DMSO acts on these signaling pathways to suppress inflammatory cytokine/chemokine production. Although DMSO induces the differentiation of B16/F10 melanoma cells *in vitro*, topical administration of DMSO to mice subcutaneously implanted with B16 melanoma cells was ineffective at reducing tumor growth, DMSO was also found to block mouse macrophages from polarizing to either an M1- or an M2-phenotype, which may contribute to its inability to slow tumor growth. Topical administration of DMSO, however, significantly mitigated K/BxN serum-induced arthritis in mice, and this was associated with reduced levels of pro-inflammatory cytokines in the joints and white blood cell levels in the blood. Thus, while we cannot confirm the efficacy of DMSO as an anti-cancer agent, the use of DMSO in arthritis warrants further investigation to ascertain its therapeutic potential.

## Introduction

Dimethyl sulfoxide (DMSO, i.e., (CH_3_)_2_SO)), by virtue of its one highly polar sulfinyl group and two non-polar methyl groups, is considered an excellent solvent, capable of dissolving many polar and non-polar compounds [1,2]. It is widely available and inexpensive because it is easily generated from dimethyl sulfide (DMS), a byproduct of the pulp and paper industry [3]. DMSO has also been used for many years to cryopreserve cells for both research and clinical applications since it prevents ice crystal formation and thus reduces cell death [4]. Its medicinal use was first promoted by Stanley Jacob, who reported in 1964 that DMSO easily penetrates the skin and carries small molecules through biological membranes [5]. Since then, a large number of publications have reported a wide range of other biological activities that suggest the potential use of DMSO as a versatile pharmacotherapy agent in a variety of medical conditions ranging from acute musculoskeletal disorders, arthritis, scleroderma, headaches and cancer [6–9]. In 1965, however, the FDA banned all clinical trials involving DMSO because it was found to cause changes in the refractive index of the lens in the eyes of a number of animals [10]. Although the regulatory restriction to conduct clinical trials on DMSO was lifted in 1980, the feverish research interest in DMSO seen in the 1960s has not been rekindled [11,12]. As a result, there have been very few recent studies to validate its safety and efficacy in various clinical settings.

Currently, the only US FDA approved medical use for DMSO is for the treatment of interstitial cystitis, via intravesicular administration of a 50% DMSO solution [2]. The FDA is leery of extending its use to other indications and lists it as a “fake cancer cure” [13]. Nonetheless, a quick internet search for “medicinal uses of DMSO” reveals that it is currently being used extensively, orally, topically and even intravenously, as an alternative treatment for a wide variety of conditions, including pain, wound healing, inflammatory disorders, viral infections and cancer [14]. Given its wide, unsanctioned use today and a paucity of data concerning its safety and efficacy, we have, herein, undertaken studies to evaluate DMSO as an anti-inflammatory agent using a novel whole human blood assay designed to measure the ability of blood cells, *ex vivo*, to respond to a bacterial and viral challenge. In addition, we have investigated the mechanism(s) by which DMSO modulates the production of pro-inflammatory cytokines/chemokines from human monocytes, the predominant cell in human blood that secretes these inflammatory mediators. Lastly, given its promotion as an alternative medicine for both cancer [15] and inflammatory disorders [16,17] we examined its effects *in vivo*, via topical application to mouse models of human melanoma and rheumatoid arthritis.

## Materials and Methods

### Reagents

The IL-6 ELISA kit was from BD Biosciences (Mississauga, ON, Canada) and the human cytokine/chemokine Luminex^®^ Multiplex Immunoassay kit and One Shot^®^ INV110 chemically competent *Escherichia coli* (*E*. *coli*) was from Life Technologies (Burlington, ON). The Prostaglandin E_2_ (PGE_2_) EIA kit was from Cayman Chemical Company (Ann Arbor, MI). Antibodies for Western blotting for phospho-JNK (#9255), phospho-p38 (#9212), and phospho-ERK1/2 (#9106S), and the MEK inhibitor U0126, were from Cell Signaling Technology (Beverly, MA, USA). Antibodies recognizing phospho-Akt (44-621G) were from Invitrogen/Life Technologies (Grand Island, NY, USA) and for Grb2 (sc-255) from Santa Cruz Biotechnology (Santa Cruz, CA. USA). DMSO (D8418), DMSO_2_ (M81705), and DMS (274380) were from Sigma-Aldrich (St Louis, MO). The NF-κB inhibitor Bay11-7082 (Bay11), the p38 inhibitor SB203580, and the PI3K inhibitor LY294002 were from Calbiochem (San Diego, CA), while the JNK inhibitor SP600125 was from EMD Millipore (Etobicoke, ON). Herpes simplex virus-1 (HSV-1) G207 was from W.J. Ammonium chloride solution, EasySep^™^ Human Monocyte Enrichment Kit without CD16 depletion, and RPMI 1640 culture media were from StemCell Technologies (Vancouver, Canada). Human AB serum was from Innovative Research (Novi, MI, USA). The melanoma B16/F10 cell line was a kind gift from Dr. Youwen Zhou of the Dermatology department at the University of British Columbia (UBC).

### Mice

Mice were bred in-house and boarded in specific pathogen free facilities at the Animal Resource Centre, BC Cancer Research Centre or the Biomedical Research Centre at UBC. All experiments and protocols were approved by and performed according to the requirements of the Canadian Council on Animal Care (CCAC) and the University of British Columbia Animal Care Committee (protocol # A14-0175 (GK) and #A14-0115 (KM)). KRN mice (generously provided by Dr. David M. Lee from Brigham and Women’s Hospital and Harvard Medical School, Boston, MA) were bred with NOD-SCID IL2Rγ^-/-^ mice (JAX #005557) to yield K/BxN mice. K/BxN mice were sacrificed at 12 weeks of age to harvest arthritogenic K/BxN serum by cardiac puncture. Eight to 10 week old C57BL/6J (JAX#000664) (B6) were used for all other mouse experiments unless otherwise stated.

### Human Whole Blood Assay

All experiments performed on human blood were reviewed and approved by the University of British Columbia Clinical Research Ethics Board (#H12-00727). Human blood was collected by phlebotomists from volunteers, who gave written consent to participate in this study, into endotoxin-free, heparin-coated tubes. Fifty microlitres of blood was aliquoted into round bottom 96 well plates, to which 10 μL of DMSO, diluted in PBS, was added or not. After 15 min of incubation at 37°C in a low oxygen (5% O_2_) incubator, inflammatory responses were initiated by the addition of *E*. *coli* (10^5^/mL), or HSV-1 (MOI = 2), (total volume = 70 μL/well), followed by incubation for 7 h at 37°C. 100 μL of PBS was then mixed well with the blood samples and the plate centrifuged at 335 x *g* using an SX4750 Rotor in an Allegra X-12R centrifuge for 5 min. Supernatants were immediately frozen at -80°C for subsequent ELISAs and Luminex analyses.

### Measurement of Cytokine, Chemokine and PGE_2_ Levels

IL-6 levels in plasma were determined by ELISA, according to the manufacturer’s instructions and results are expressed as a percent of the IL-6 level triggered by non-DMSO-treated blood samples in the absence (0%) or presence (100%) of *E*. *coli* or HSV-1. PGE_2_ in plasma was quantified using a PGE_2_ EIA kit according to the manufacturer’s instructions. Luminex analyses to simultaneously quantify the levels of IL-1β, G-CSF, IL-10, IL-13, IL-6, IL-17, MIP-1α (CCL3), VEGF, IFNγ, IL-12p70, IFNα, IL-1RA, TNF-α, IL-4 and IL-8 (CXCL8) were performed using a Luminex 100 Bio-Plex Microplate Analyzer (Bio-Rad Laboratories, Hercules, CA). Acquired fluorescence was analyzed by the Bio-Plex Manager^™^ version 6.0 (Bio-Rad Laboratories).

### DMSO, DMS and DMSO_2_

To compare the relative efficacy of DMSO with its metabolites, DMS and DMSO_2_, on modulating inflammatory responses, the 3 compounds were analyzed in the whole blood assay as described above. The three compounds were dissolved in autologous plasma instead of PBS to increase the solubility of DMS in the whole blood assay. Also, to prevent the volatile DMS from affecting the evaluation of other treatments (DMSO and DMSO_2_), the 96 well plate was sealed with a plate sealer during the 7 h incubation period.

### Cell Viability Assays

In parallel studies, whole human blood samples (70 μL/well) were collected at the end of the 7 h incubation period to determine the effect of DMSO, DMSO_2_ and DMS on cell viability using propidium iodide staining. Specifically, following the 7 h incubation assay, red blood cells were lysed by the addition of 2.5 mL of ammonium chloride solution (StemCell Technologies) in polystyrene flow tubes. After 15 min of incubation on ice, cells were pelleted by centrifugation at 335 x *g* for 5 min, followed by a wash step with cold PBS containing 2% FBS and 0.05% sodium azide (HFN). Cells were then stained with 1 μg/mL propidium iodide in 300 μL of HFN and subjected to flow cytometric analysis using a FACSCalibur (Becton Dickinson).

### Fractionation of Blood Cells

Cells were fractionated from whole blood by Ficoll density gradient centrifugation. Neutrophils were recovered from the granulocyte layer, which was subjected to ammonium chloride lysis to remove red blood cells. Peripheral blood mononuclear cells (PBMCs) were collected from the buffy coat, reconstituted in 50% autologous plasma and seeded at 4.5x10^4^ cells/50 μL into flat bottom 96 well plates. Monocytes were obtained from PBMCs by adherence of the cell mixture to a flat bottom 96 well plate for 1h in a 37°C incubator. The non-adhering lymphocytes were collected and seeded in 50% autologous plasma in separate wells. The monocytes, lymphocytes and neutrophils were then challenged with *E*. *coli* as described above. After 7 h of incubation, the plates were centrifuged as above and the supernatants collected for IL-6 analysis.

### Cell Signaling in Human Monocytes

White blood cells collected by apheresis from G-CSF-mobilized normal stem cell donors were obtained from the Stem Cell Assay Laboratory/Hematology Cell Bank of the British Columbia Cancer Agency. Monocytes from these apheresis samples were then isolated using an EasySep kit according to the manufacturer’s instructions and assessed as >92% CD14^+^ by flow cytometry or by adherence. They were seeded at 2 x 10^6^ cells/well in flat bottom 12 well plates in serum-free RPMI 1640 medium. After 2 h at 37°C, the serum-free medium was removed and 1 mL RPMI 1640 medium ± DMSO (at a final concentration of 2%) was added to the cells. After 15 min at 37°C, the cells were challenged with *E*. *coli* (at a final concentration 2x10^5^/mL) for 15 and 30 min. Whole cell lysates were prepared for Western blotting by washing cells once with cold PBS followed by the addition of SDS sample buffer (1x) to the cell pellets. The samples were sheared with a 26G needle prior to boiling for 1 min. Whole cell lysates in SDS sample buffer were loaded onto 10% polyacrylamide gels. Upon transfer to PVDF membranes, separated proteins were probed for p-Akt, p-p38, p-JNK, p-ERK1/2 and Grb-2. Primary antibodies were used at 1/1000 dilution, and Grb-2 was used as a loading control.

To establish the importance of specific cell signaling pathways to the production of pro-inflammatory cytokines from LPS-stimulated human monocytes, the NF-κB inhibitor, Bay11, the p38 inhibitor, SB203580, the PI3K inhibitor, LY294002, and the JNK inhibitor, SP 600125, were added to adherent human monocytes cultured in RPMI 1640 containing 10% AB+ serum in flat bottom 96 well plates. After 15 min at 37°C, the cells were challenged with *E*. *coli* (final concentration 10^5^/mL) for 7 h. Supernatants were recovered and IL-6 levels determined by ELISA as above.

### Differentiation of B16/F10 Cells

Mouse melanoma B16/F10 cells were seeded at 2.5 x 10^4^ cells/10 mL media in 100 mm culture plates. After overnight incubation, DMSO was added at a final concentration of 0, 1, and 1.5% and the plates incubated for 6 days at 37°C. The cells were harvested by trypsinization, washed once with PBS, and cell numbers adjusted to 7x10^5^ cells/mL with 1M NaOH containing 10% DMSO. The melanin content was extracted as described in [18] using a modified protocol in which the cells were heat-treated at 80°C for 2 h, followed by centrifugation at 16,060 x *g* in a Heraeus Biofuge pico microcentrifuge. Supernatants were recovered and optical densities measured at 400 nm in a Multiscan plate reader (Labsystem, Finland).

### *In Vivo* Melanoma Assay

Logarithmically growing (<50% confluency) mouse melanoma B16/F10 cells were harvested by trypsinization, washed twice with ice-cold HBSS and resuspended in cold PBS at 5x10^6^ cells/mL [19]. They were then subcutaneously implanted (5x10^5^ in 100 μL PBS) into the shaved backs of 16 isoflurane anesthetized, female C57BL/6 mice. The treatment group received topical administration of 70% DMSO dissolved in sterile water (i.e., 1 mL in a 2”x 2” Dermacea gauze sponge, folded in four), 4 times/day, starting immediately after tumour implantation (day 0). The control group received topical administration of sterile water instead of 70% DMSO. The gauze pads soaked in either 70% DMSO or water were manually placed on the shaved backs of the mice for 1 min per treatment period. To prevent mice from licking the DMSO off each other, individual mice were isolated for 30 min while the DMSO dried by placing them in custom-made cages with plastic partitions that separated the cage into quadrants after every treatment. The mice were then placed back in their original cages. Tumor sizes were measured daily with manual calipers, and tumor volumes calculated using the formula (Length × Width × Height) × π/6. All mice were euthanized 12 days after tumor implantation and blood was obtained via cardiac puncture to determine plasma IL-6 levels. Tumors were excised and weighed.

### Macrophage Polarization Assays

Murine bone marrow derived macrophages (mФs) were polarized to classically (M1) or alternatively (M2) activated mФs according to Weisser et al [20]. Briefly, bone marrow cells were aspirated from 8–12 week old C57Bl6 mice and differentiated in GM-CSF (10 ng/mL) or M-CSF (12 ng/mL)-containing Iscove’s Modified Dulbecco’s Medium in the presence of 150 μM monothioglycerol (MTG) and 10% FCS. The cells were differentiated for 10 days with media changes at days 3 and 7. At day 10, cells were re-plated in a 12 well plate at 5x10^5^ cells/well and treated with DMSO at final concentrations of 0.25% to 1%. To polarize mФs to an M1 phenotype, GM-CSF-differentiated cells were stimulated with LPS at 100 ng/mL for 24 hrs, ± DMSO. For M2 skewing, M-CSF differentiated mФs were treated with IL-4 (10 ng/mL) for 48 hrs ± DMSO. Cells were then washed once with PBS, resuspended in SDS sample buffer (1x) as described above and subjected to Western blot analysis. M2-skewed mФ lysates were probed for Arg-1 and Ym-1 while M1 skewed mФ lysates were probed for iNOS. Grb-2 was used as a loading control.

Nitric oxide (NO) levels in M1 skewed mФs were measured by mixing 100 μL of cell supernatant with 100 μL of Griess reagent. The absorbances of the samples were measured at 540 nm using a Sunrise^™^ Tecan spectrophotometer (Switzerland)

### *In Vivo* Arthritis Model

Arthritis was induced in 10–12 week old male C57BL/6 mice by two intraperitoneal (IP) injections of 100 μL of K/BxN serum (generated in-house) on days 0 and 2 [21]. One day (24 h) before the initial IP injection of K/BxN serum, the hind paws of the mice were dipped for 30 s per paw in a 70% DMSO/30% autoclaved water solution (or autoclaved water alone as a control). Hind paws were fully immersed up to the ankle fur line using a 50 mL falcon tube lid filled to capacity. The thickness of hind ankle and fore paws was measured daily using a dial thickness gauge (Mitutoyo 7305, with 0.01 mm graduations from KBC Tools, Vancouver, BC). At the same time, a clinical score for each paw was assigned as previously described [22] with slight modifications: 0 = no sign of swelling, 1 = mild swelling and/or redness, 2 = swelling of ankle and midfoot/paw, 3 = swelling involving the whole joint and midfoot/paws, and 4 = severe swelling encompassing the ankle, foot/paw and fingers/toes. Daily clinical score and ankle thickness measurements of the hind and/or fore paws were summed up together and compared to baseline scores/measurements. On day 7, mice were euthanized by CO_2_ asphyxiation. Plasma was collected immediately after sacrifice by cardiac puncture using a syringe containing 20 μL of 0.5 M EDTA. The skin covering the joints was removed and whole joints were snap-frozen in dry ice and stored at -80°C for later analysis or fixed for histology as described below.

### Quantitative PCR

The gene expression levels of IL-6, TNFα, IL-1β, CXCL1, and CXCL2 in the joints were determined by real time qPCR. RNA isolation from crushed tissue joints was performed after bead mill homogenization of the joints in 1 mL of Trizol. RNA was quantified using a ND1000 nanodrop spectrophotometer and cDNA was synthesized from 2 μg of RNA using a High Capacity cDNA Reverse Transcription kit (Applied Biosystems). cDNA was mixed with SYBR FAST (qPCR kit, KAPA Biosystems) and primers specific to the genes of interest (listed in Table 1). PCR amplification was performed on an ABI 7900 HT instrument (Applied Biosystems, Foster City, CA, USA). The mRNA expression level of the genes of interest was expressed relative to *Gapdh* transcript levels.

**Table 1:** Primers for real time gene expression analysis.

Gene name	Forward (5’-3’)	Reverse (5’-3’)
*Gapdh*	CGTGCCGCCTGGAGAAACC	TGGAAGAGTGGGAGTTGCTGTTG
*Il6*	TAGTCCTTCCTACCCCAATTTCC	TTGGTCCTTAGCCACTCCTTC
*Tnfa*	CATCTTCTCAAAATTCGAGTGACAA	TGGGAGTAGACAAGGTACAACCC
*Il1b*	GCAACTGTTCCTGAACTCAACT	ATCTTTTGGGGTCCGTCAACT
*Cxcl1*	CTGGGATTCACCTCAAGAACATC	CAGGGTCAAGGCAAGCCTC
*Cxcl2*	AGTGAACTGCGCTGTCAATGC	CCATCCAGAGCTTGACGGTGAC

### Determination of DMSO levels

The DMSO concentration in plasma and joints was quantified using HPLC/MS. Deuterated DMSO (d_6_-DMSO) was used as an internal standard. Plasma samples were diluted with acetonitrile containing internal standard, vortexed, centrifuged for 5 min at 15,000 x g and supernatants transferred to LC vials. DMSO from crushed joints was extracted by the addition of 9 volumes of acetonitrile to the joints. The samples were vortexed, incubated at 4°C overnight, and centrifuged at 16,060 x *g* in a Heraeus Biofuge pico microcentrifuge for 10 min. Supernatants were analyzed for DMSO content in an identical fashion to that performed for plasma samples. Samples were analyzed with a Waters Acquity LC coupled to a Waters Quattro Premier using a BEH Amide, 1.7μ column (Waters). Water (0.1% formic acid (FA)) and ACN (0.1% FA) were LC solvents A and B, respectively. Gradient runs were performed as follows: 94% B at 0–1.1min; 94–50% B at 1.1–1.2min; 50% B at 1.2–2.6min; 50–94% B at 2.6–2.7min with total run length 5.5min at 0.4mL/min. All MS data were collected in ES+ at unit resolution with the following parameters: capillary, 3.5kV; extractor and RF lens, 5V and 0.2V; source and desolvation temperatures, 120°C and 350°C; desolvation and cone (N_2_) flow, 1000L/hr and 50 L/hr; collision gas (Ar) flow, 0.17ml/min (8.1e^-3^ m bar). Detection was by multiple reaction monitoring with m/z 79>64 for DMSO and m/z 85>67 for d_6_-DMSO (35V/14V cone/collision volt for both) with 0.1sec dwell each and 1.06 min retention time (RT) for both. Quantitation was via the AUC ratio of DMSO/d6-DMSO with a linear fit through calibration data (R^2^>0.99); accuracy/precision > ±15% (n = 3) above 50 nM.

### Blood Differential Analysis and Joint Lavage

To identify the effect of topical administration of DMSO on circulating blood cells and immune cells recruited to the joints, blood samples were obtained by cardiac puncture, and joint lavage was carried out to recover immune cells. Briefly, the hind paws of 18 week old female C57BL/6NHsd were immersed in 100% DMSO or distilled water for 1 min, twice daily, beginning 48 h prior to K/BxN serum injection (100 μL IP at day 0 and day 2). DMSO or water-treated mice that were IP injected with PBS instead of K/BxN serum served as controls. Mice were sacrificed at day 3 and exactly 300 μL blood was collected via cardiac puncture into EDTA-containing tubes (20 μL of 0.5 M EDTA). Complete blood count of blood was performed using a scil Vet animal blood counter hematology analyzer (Viernheim, Germany). The immune infiltrate in the joints was determined by joint lavage as described [22] The cells in the joint lavage were subsequently stained with a viability marker (7AAD) (ThermoFisher Scientific, Waltham, MA, USA), CD45.2-eFluor450 (clone 104 from ebioscience, San Diego, CA, USA), 7/4-FITC (ab53453 from Abcam, Toronto, ON, Canada) and Gr-1-PE (clone RB6-8C5 from AbLab.ca, Vancouver, BC) to identify polymorphonuclear (PMN) cells, by flow cytometry (LSR II, BD Bioscience, San Jose, CA, USA) using FlowJo software (FlowJo, Ashland, OR, USA) for data analysis. Pre-calibrated counting beads (ThermoFisher Scientific) were added to the final flow cytometry sample tubes to determine the total live cells recovered from the joints.

## Results

### DMSO is Anti-inflammatory in a Whole Human Blood Assay

Given DMSO’s rapid uptake into the blood stream [23] we first examined its effects on whole human blood, optimized, *ex vivo*, to mimic *in vivo* conditions as closely as possible. Specifically, we used whole human blood rather than isolated PBMCs, which have been used extensively in the past for cytokine/chemokine expression studies [24,25], in order to eliminate potential artifacts that might result from lengthy isolation procedures and to mimic *in vivo* conditions more closely since red blood cells, granulocytes and plasma are still present. In addition, we have incubated whole blood samples under physiological levels of oxygen (5% rather than 21%) at 37°C for 7 h (to minimize changes in the properties of the blood cells that might occur with longer *in vitro* incubation times). Preliminary studies in our lab have shown that this time period is sufficient to detect both early and late secreted cytokines and chemokines from leukocytes in whole blood assays (data not shown). We have also challenged our whole blood samples with either intact bacterial (*E*. *coli*), or virus (HSV-1), rather than specific Toll like receptor (TLR) agonists like lipopolysaccharide (LPS), since we considered intact pathogens more representative of *in vivo* infections.

**Fig 1:**
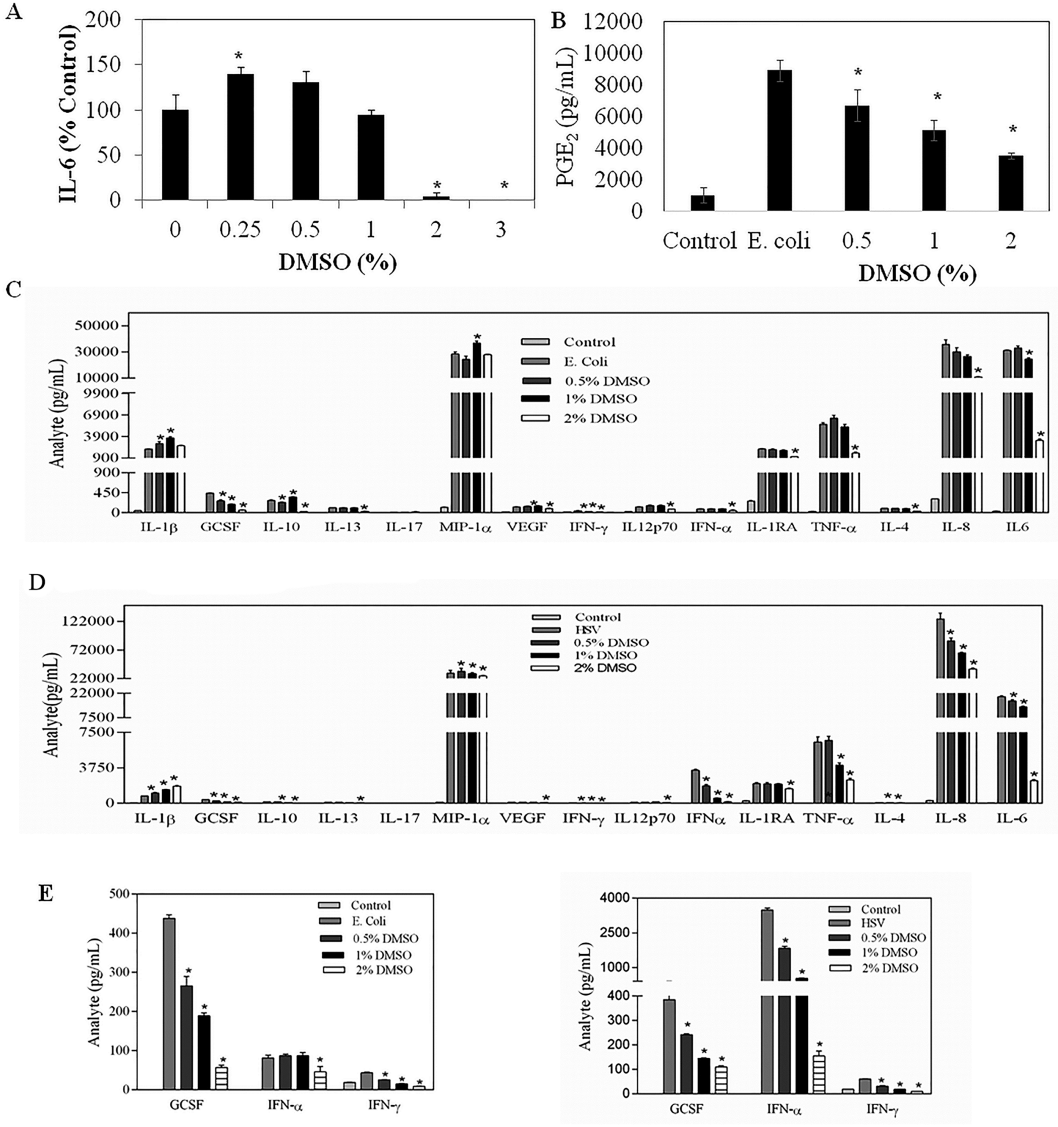
The effect of DMSO on the (A) IL-6, (B) PGE_2_ and (C) 15 cytokine/chemokine secreted in a whole human blood assay in response to *E. coli* or (D) HSV stimulation. * denotes significant difference (P < 0.05) from non-DMSO treated, *E. coli* challenged or HSV-stimulated blood.

As shown in Fig 1A, DMSO dose response studies with whole human blood samples from healthy volunteers, challenged with *E*. *coli*, revealed that final concentrations of 0.25% DMSO modestly but consistently increased IL-6 production whereas, concentrations of 1% and greater were inhibitory. In addition, DMSO at 0.5% and higher significantly suppressed PGE_2_ secretion from *E*. *coli*-stimulated whole blood cells (Fig 1B), confirming the anti-inflammatory activity of DMSO in a whole human blood assay.

### DMSO Modulates both Bacterial and Viral Stimulated Cytokines

To expand on these findings we used Luminex technology to determine the effect of DMSO on the *E*. *coli*-stimulated secretion of a total of 15 cytokines/chemokines from normal human whole blood cells. As shown in Fig 1C, 2% DMSO significantly reduced the levels of 13 of the cytokines/chemokines. The two notable exceptions were interleukin-1β (IL-1β), the only analyte within this panel that is cleaved/matured in the inflammasome [26] and mФ inflammatory protein (MIP-1α), the only analyte tested that is a member of the C-C family of pro-inflammatory chemokines [27]. Also of note, lower concentrations (0.5% and/or 1%) of DMSO actually promoted the expression of these two analytes, suggesting a potentially distinct effect of DMSO on different cell signaling pathways. Importantly, some analytes were far more sensitive to DMSO-induced inhibition. G-CSF and IFNγ, for example, were extremely sensitive, showing substantial reductions at 0.5% DMSO (see below).

We also used Luminex technology to ascertain the effect of DMSO on the secretion of these 15 cytokines/chemokines from virally (i.e., HSV-1) stimulated normal human whole blood cells. As shown in Fig 1D, IL-1β and MIP1α were, once again, not inhibited by 2% DMSO and, in fact, IL-1β levels were dose dependently increased with DMSO. As well, G-CSF and IFNγ were extremely sensitive to inhibition by DMSO. IFNα levels were stimulated to far higher levels with HSV-1 than *E*. *coli*, which is expected since this cytokine is typically secreted during viral infections [28]. This too, was markedly inhibited with only 0.5% DMSO. The effect of DMSO on these analytes, triggered by either *E*. *coli* or HSV-1 is more clearly shown in Fig 1E, where we have expanded the Y ordinate.

### Cytotoxicity of DMSO

**Fig 2:**
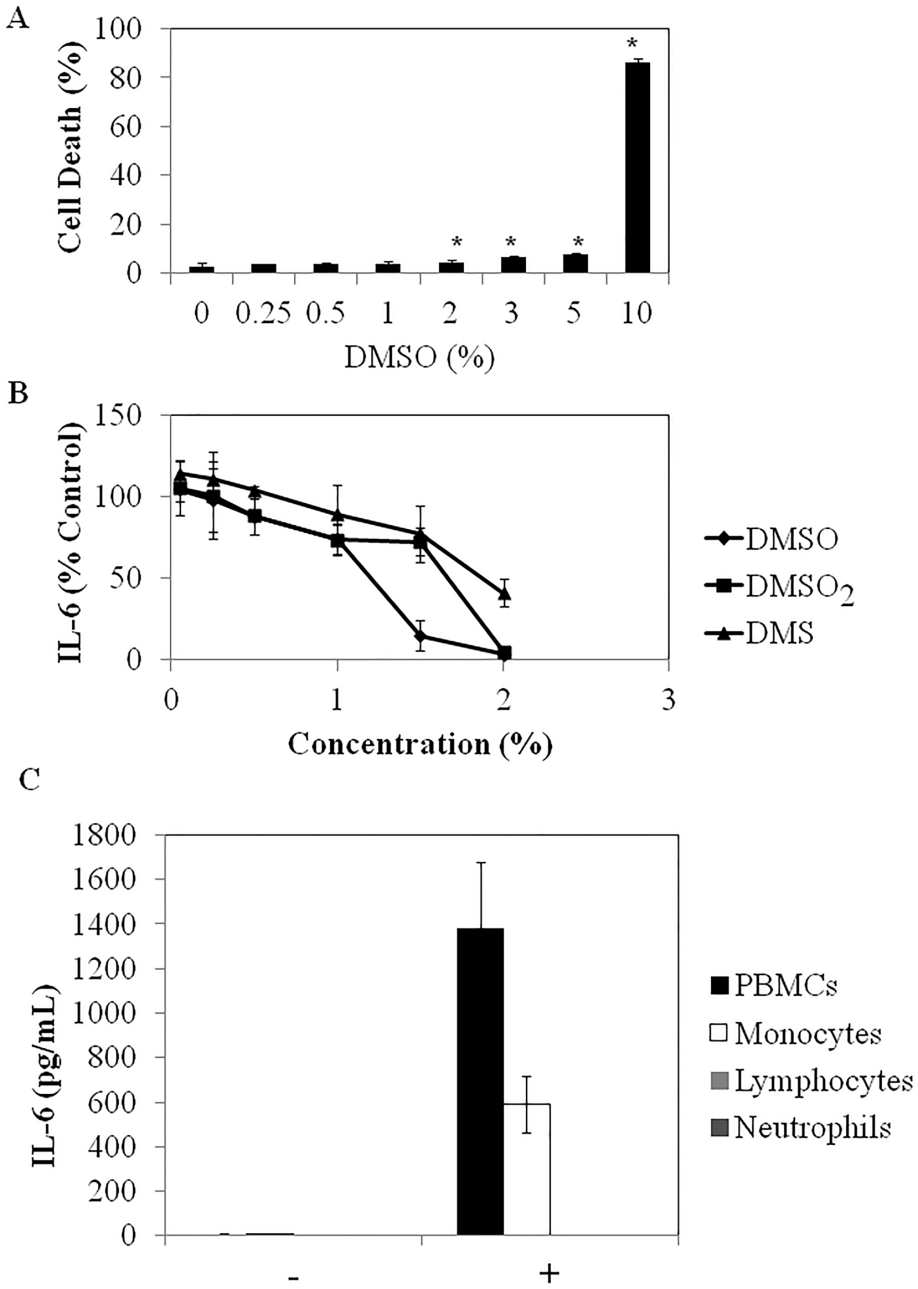
**(A)** The dose dependent effect of DMSO on human blood cell viability. After 7 h of incubation, red blood cells were lysed with ammonium chloride and the white blood cells stained with propidium iodide and analysed by flow cytometry. *denotes significant difference (P < 0.05) from non-DMSO treated, *E*. *coli* challenged blood. **(B)** The effects of DMSO, DMS and DMSO_2_ on *E*.*coli*-stimulated production of IL-6 in the human whole blood assay. **(C)** A comparison of *E*. *coli*-induced cytokine/chemokine production from monocytes, lymphocytes and granulocytes. Data are expressed as mean ± SD of triplicate determinations from 1 experiment representative of 2 or 3 independent experiments with blood from different healthy donors. (-) and (+) refers to treatment of cells without and with *E*. *coli*, respectively.

To determine if the DMSO-induced reductions in cytokine/chemokine production were due to cytotoxic effects on blood cells, its effect on blood cell death was determined by propidium iodide staining. As shown in Fig 2A, 7 h of treatment with DMSO was not cytotoxic to blood cells when tested at final concentrations up to 1% and only slightly, but significantly (P<0.05) cytotoxic at 2% to 5%, where it reduced cell viability by 4.7 to 7.8%, respectively. However, when the concentration of DMSO was increased to 10%, cell death was dramatically increased to 86.4% which also corresponds to the concentrations at which extensive hemolysis was observed (data not shown), in keeping with previous studies [29]. Of note, when the effects of DMSO on the viability of white blood cell (wbc) subtypes was examined, monocytes were more sensitive than granulocytes or lymphocytes, with viability beginning to decrease at a final DMSO concentration of 1%, whereas granulocyte and lymphocyte viability did not significantly decrease until levels between 5 and 10% (Table 2). At 10% DMSO, all wbc subsets were drastically decreased, which corresponds to the overall PBMC toxicity observed with PI staining and flow cytometry (Fig 2A). These results suggest that DMSO has a narrow therapeutic concentration range between efficacy as an anti-inflammatory agent and cytotoxicity to the cells that produce the bulk of the cytokines/chemokines (i.e., monocytes, see below).

**Table 2:** The effect of DMSO on white blood cell subtypes stimulated with *E. coli* in the human whole blood assay.

	Control	E. coli	0.25%	0.5%	1%	2%	3%	5%	10%
**Granulocytes**	56.2 ± 1.1	55.2 ± 2.3	52.8 ± 1.7	47.8 ± 1.4*	48.7 ± 2.2*	46.9 ± 7.8	52.5 ± 3.0	58.2 ± 0.7	27.1 ± 1.2*
**Lymphocytes**	32.1 ± 0.4	34.6 ± 1.9	36.6 ± 1.5	41.0 ± 1.2*	39.1 ± 1.6*	43.7 ± 8.1	36.6 ± 2.3	27.8 ± 0.8*	15.6 ± 1.0*
**Monocytes**	7.9 ± 0.3	6.8 ± 0.3	7.8 ± 0.5*	7.8 ± 0.7*	6.1 ± 0.2*	3.7 ± 0.5*	1.6 ± 0.2*	2.0 ± 0.3*	1.8 ± 1.3*

* denotes significant (P <0.05) difference between DMSO-treated and untreated cells. Data are expressed as % of total cells mean ± SD) of triplicates from a representative experiment from two independent studies. The cells were subtyped via flow cytometry, based on their forward and side scatter properties.

### The Effect of DMSO Metabolites on IL-6 Production

DMSO generates two major metabolites *in vivo*, the highly volatile, malodorous DMS, responsible for the garlic taste that occurs after DMSO administration and the oxidized DMSO derivative, DMSO_2_ [16]. A previous study examining the kinetics of their appearance and clearance in humans revealed that neither of these derivatives ever reaches the blood concentration of DMSO [23]. Nonetheless, to determine if they might play a role in the anti-inflammatory properties attributed to DMSO and we compared their ability to modulate IL-6 production from E. coli stimulated human blood. As shown in Fig 2B, DMSO_2_ and DMS were less effective than DMSO in reducing *E*. *coli*-induced IL-6 production. Of note, at concentrations less than 2%, DMS induced the most hemolysis as well as the most wbc death, while DMSO_2_ was similar to DMSO in its induction of cell death in the 7 hr blood assay, as demonstrated by propidium iodide staining and flow cytometry (data not shown).

### The Effect of DMSO on Cell Signaling Pathways

**Fig 3:**
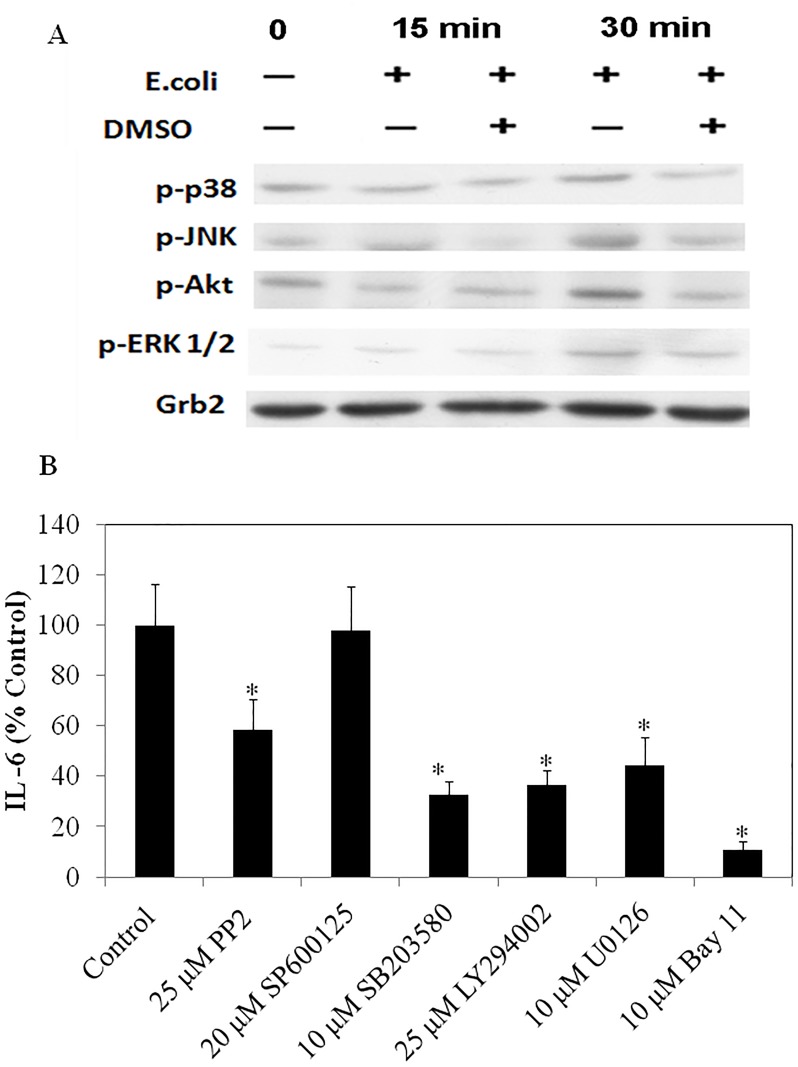
**(A)** The effect of DMSO on modulating the activation of cell signaling pathways in response to *E*. *coli-* stimulation of human blood monocytes. **(B**) The effect of cell signaling inhibitors on IL-6 production form *E*. *coli*-challenged human monocytes. Inhibitor concentrations were chosen based on the lowest levels that markedly inhibited their pathways. *denotes significant inhibition (P<0.05) of IL-6 production from non-DMSO treated, *E. coli* challenged monocytes.

To identify which of the three major wbc subtypes, (monocytes, lymphocytes or granulocytes), was responsible for the majority of the inflammatory cytokines/chemokines produced in response to *E*.*coli* challenge in the whole human blood assay, these three subtypes were separated and challenged with *E*. *coli*. As shown in Fig 2C, monocytes were the only cellular fraction that produced detectable IL-6 in response to *E*. *coli* stimulation. Luminex studies showed that this was also the case for the other 14 cytokines/chemokines (data not shown). We therefore focused on human monocytes to gain some insight into how DMSO was reducing cytokine/chemokine production. Since previous reports have shown that LPS activation of human monocytes leads to the phosphorylation of both the PI3K and the three MAPK (i.e. p38, JNK and ERK1/2) pathways [30] we first examined the effect of *E*. *coli* on these pathways in human monocytes. As shown in Fig 3A, after 30 min of E. *coli* stimulation, Akt, p38, JNK and ERK1/2 were all phosphorylated [30]. Importantly, in the presence of 2% DMSO, there was a marked reduction in the phosphorylation of all of them. We then followed up these studies with various pathway inhibitors using U0126, LY294002 and SB203580 to inhibit the ERK, PI3K and p38 signaling pathways, respectively. All appear to be involved in IL-6 production from *E*. *coli* challenged human monocytes with the exception of JNK; we saw no differences in the presence of SP600125 (Fig 3B). Of note, the NFκB inhibitor, Bay 11, also markedly inhibited IL-6 production from *E*. *coli* challenged human monocytes (Fig 3B), consistent with previous reports showing the importance of this transcription factor to inflammatory cytokine production in TLR agonist-stimulated human monocytes [30]. In addition, PP2 which is an inhibitor of the Src family was also effective in reducing IL-6 production and this appeared to be mediated through inhibition of the PI3K pathway, since PP2 reduced Akt phosphorylation, but not Erk phosphorylation (data not shown).

### DMSO Does Not Reduce the *In Vivo* Growth Rate of Melanomas

**Fig 4:**
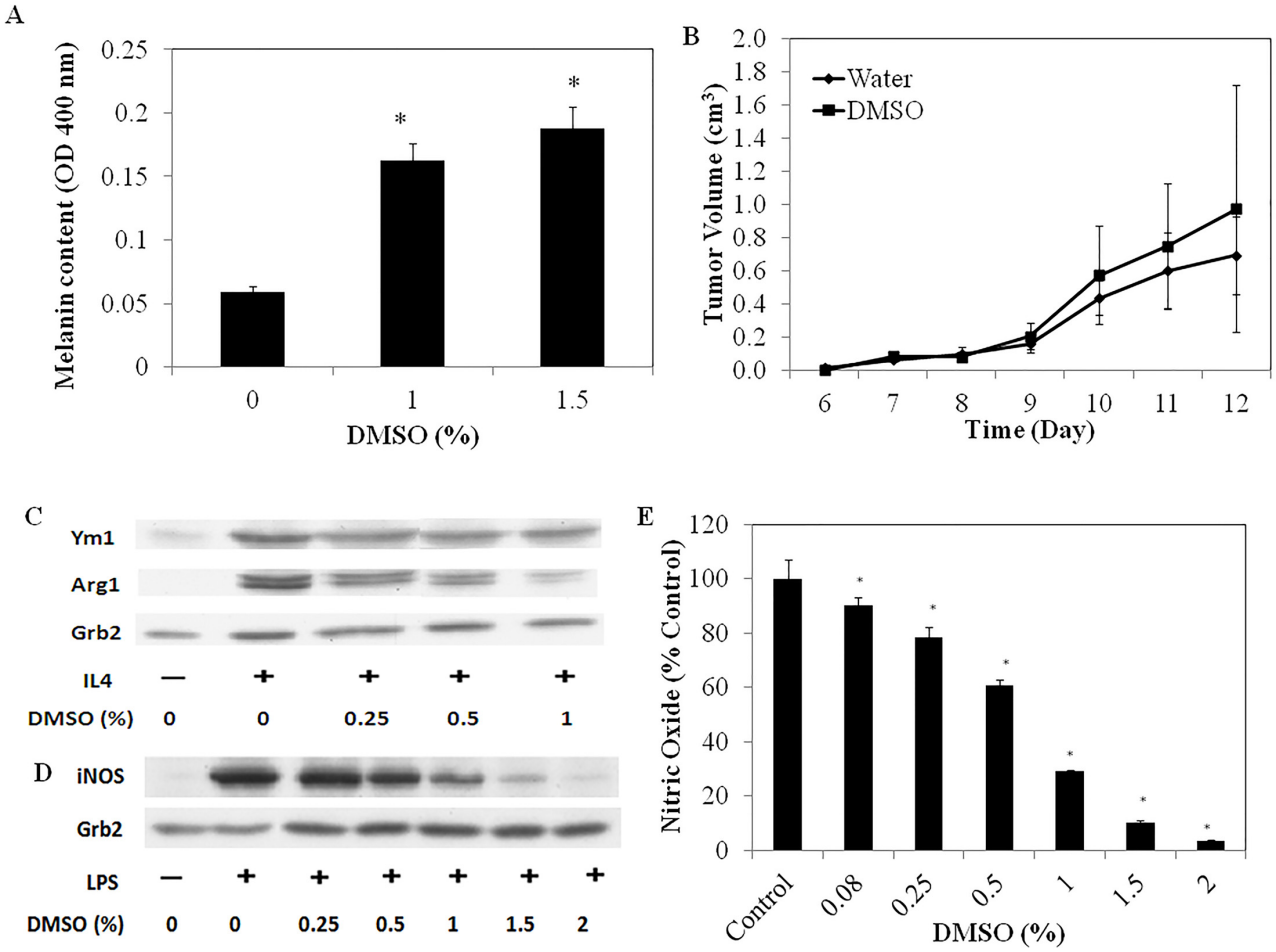
**(A)** The effect of DMSO on melanin production in B16/F10 melanoma cells. *denotes significant difference (P<0.05) in melanin content to non-DMSO treated cells **(B)** The effect of topical administration of 70% DMSO on the growth of subcutaneously implanted B16/F10 melanoma cells in C57BL/6 mice (n = 8) (**C**) The effect of DMSO on murine M2 mФ skewing in response to 10 ng/ml IL-4 measured after 72 h of incubation (**D**) The effect of DMSO in modulating iNOS expression and **(E)** NO production from M1-skewed murine mФs, measured after 24 h of incubation of cells with 100 ng/mL LPS.

DMSO is currently used as an alternative treatment for various cancers. To assess its efficacy as an anti-cancer agent we used the B16 melanoma mouse model since it is both amenable to topical treatment and potentially relevant to treating human cancers topically. Preliminary *in vitro* studies with these tumor cells demonstrated that DMSO at 1 and 1.5% reduced cell proliferation (data not shown) and, concomitantly, increased the levels of melanin in these cells, in keeping with previous reports that DMSO induces, to some extent, B16 differentiation (Fig 4A) [31].

However, even though DMSO could induce the partial differentiation of these melanoma cells, and so might be expected to slow tumor growth in the B16-implanted mice, we found that topical administration of 70% DMSO (directly to skin at the tumor site) was ineffective in altering their growth and comparable to topical administration of water alone (Fig 4B).

### DMSO Inhibits both M1 and M2 Polarization

To gain some insight into why DMSO was ineffective at slowing down melanoma cancer progression, we investigated the effect of DMSO on the ability of mФs to polarize to a healer (M2) or killer (M1) phenotype and found that DMSO, starting at 0.25% final concentrations, effectively suppressed the expression of arginase 1 (Arg-1) in IL-4-treated, bone marrow derived mФs, suggesting suppression of M2 skewing (Fig 4C). At the same concentrations, DMSO also reduced the expression of inducible nitric oxide synthase (iNOS) from LPS-treated mФs, suggesting it also suppresses M1 polarization (Fig 4D). Consistent with this, DMSO, at concentrations as low as 0.08% significantly reduced nitric oxide (NO) production from these cells (Fig 4E). To assess whether these effects were attributable to cell death, viability studies were carried out and DMSO had negligible effects on mФ viability at concentrations that inhibited skewing (data not shown). DMSO thus appears to block the polarization of mФs to either M1 or M2 mФ phenotypes.

### DMSO Reduces Arthritis

**Fig 5:**
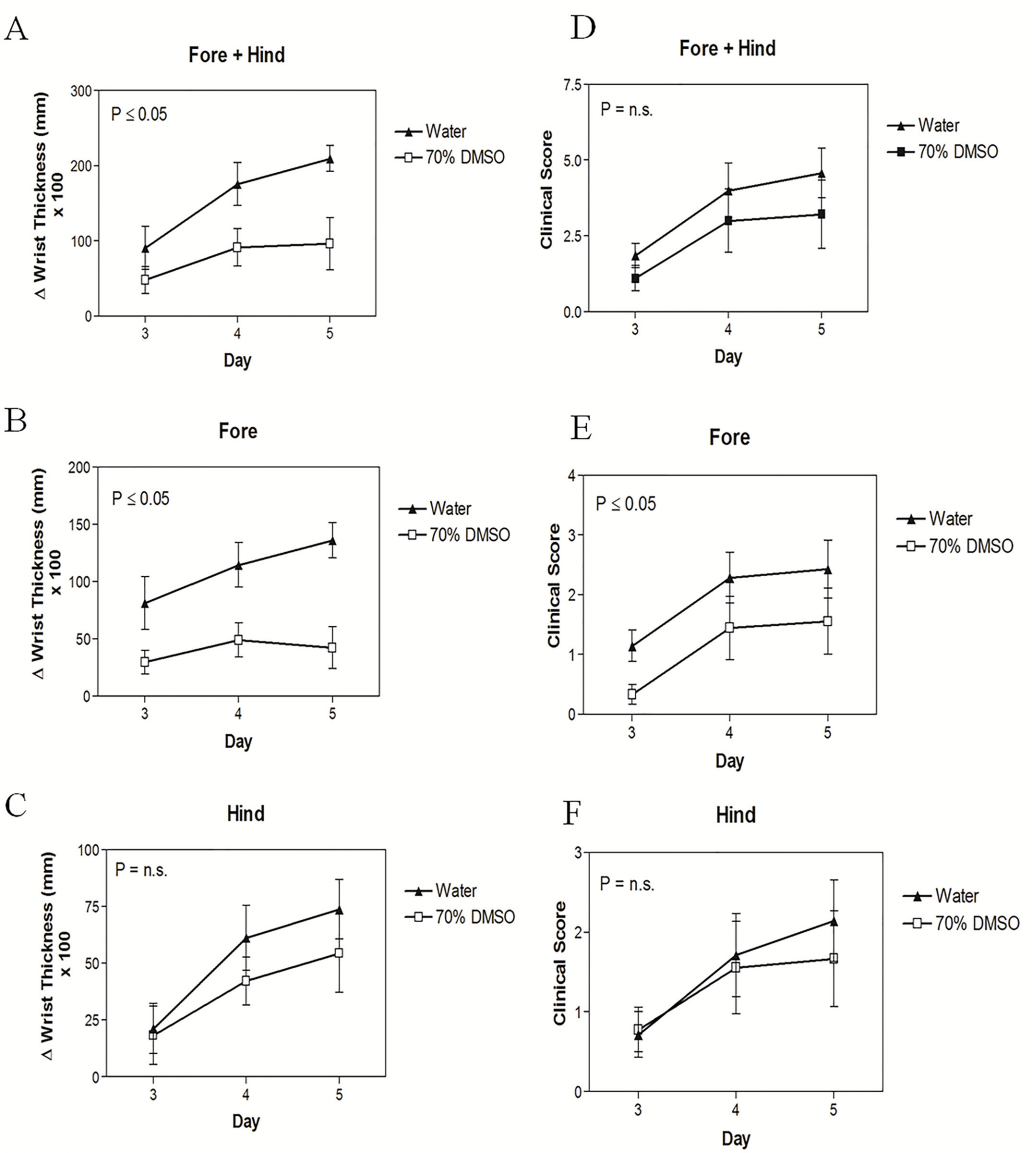
The effect of DMSO in modulating arthritis-induced swelling in the **(A)** fore, **(B)** hind and **(C)** fore + hind paws of K/BxN serum-injected C57BL/6 mice. The clinical scores of the **(D)** fore, **(E)** hind **(F)** and fore + hind paws in DMSO and water-treated mice (n = 7–9).

Since topical DMSO has been used for many years to reduce both inflammation following exercise or injury and for arthritis, we next examined its efficacy using a mouse model of arthritis. Specifically, we employed the well-known K/BxN autoimmune arthritis model in which serum containing auto-antibodies that target glucose-6-phosphate isomerase (GPI) is injected IP. These auto-antibodies trigger inflammatory responses in the joints of recipients, resembling the pathogenesis of rheumatoid arthritis [32–34]. As shown in Fig 5A, immersion of the hind paws of C57BL/6 mice into 70% DMSO twice daily was effective in reducing the overall swelling of K/BxN-induced arthritis in all hind and fore paws. Interestingly, when the effects of DMSO on hind and fore paws were evaluated separately, DMSO was found to significantly reduce the swelling of the front paws only (Fig 5B & 5C), although there was a trend for DMSO to ameliorate swelling in the hind paws as well. These results correlated with reductions in clinical scores for the DMSO treated mice (Fig 5D, 5E & 5F).

**Fig 6:**
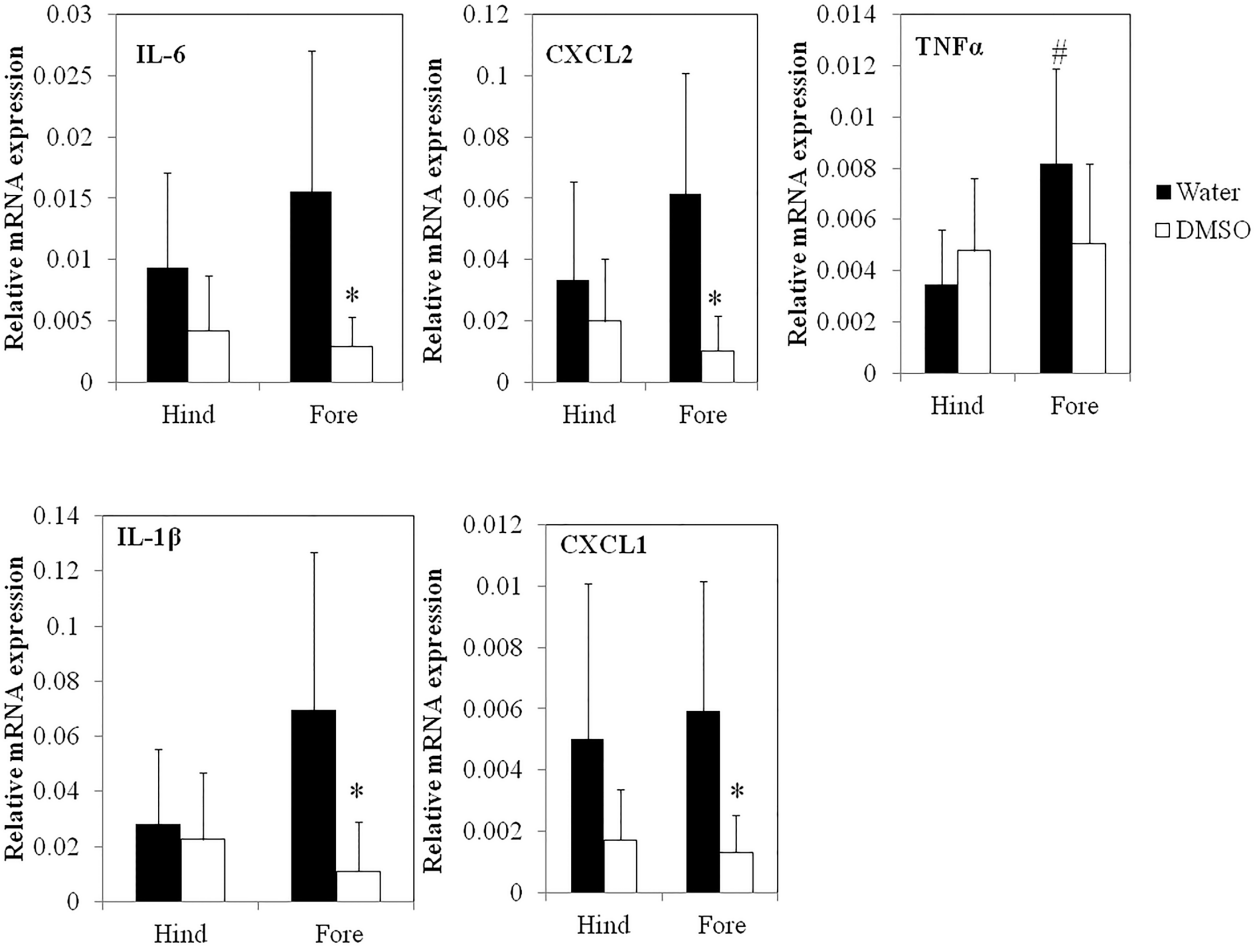
The effect of DMSO on the gene expression of pro-inflammatory cytokines in the joints of KBxN-induced arthritic mice. *denotes significant (P<0.05) difference between DMSO and water-treated joints. ^#^ denotes a significant (P<0.05) difference between the fore and hind paws of water-treated mice.

**Fig 7:**
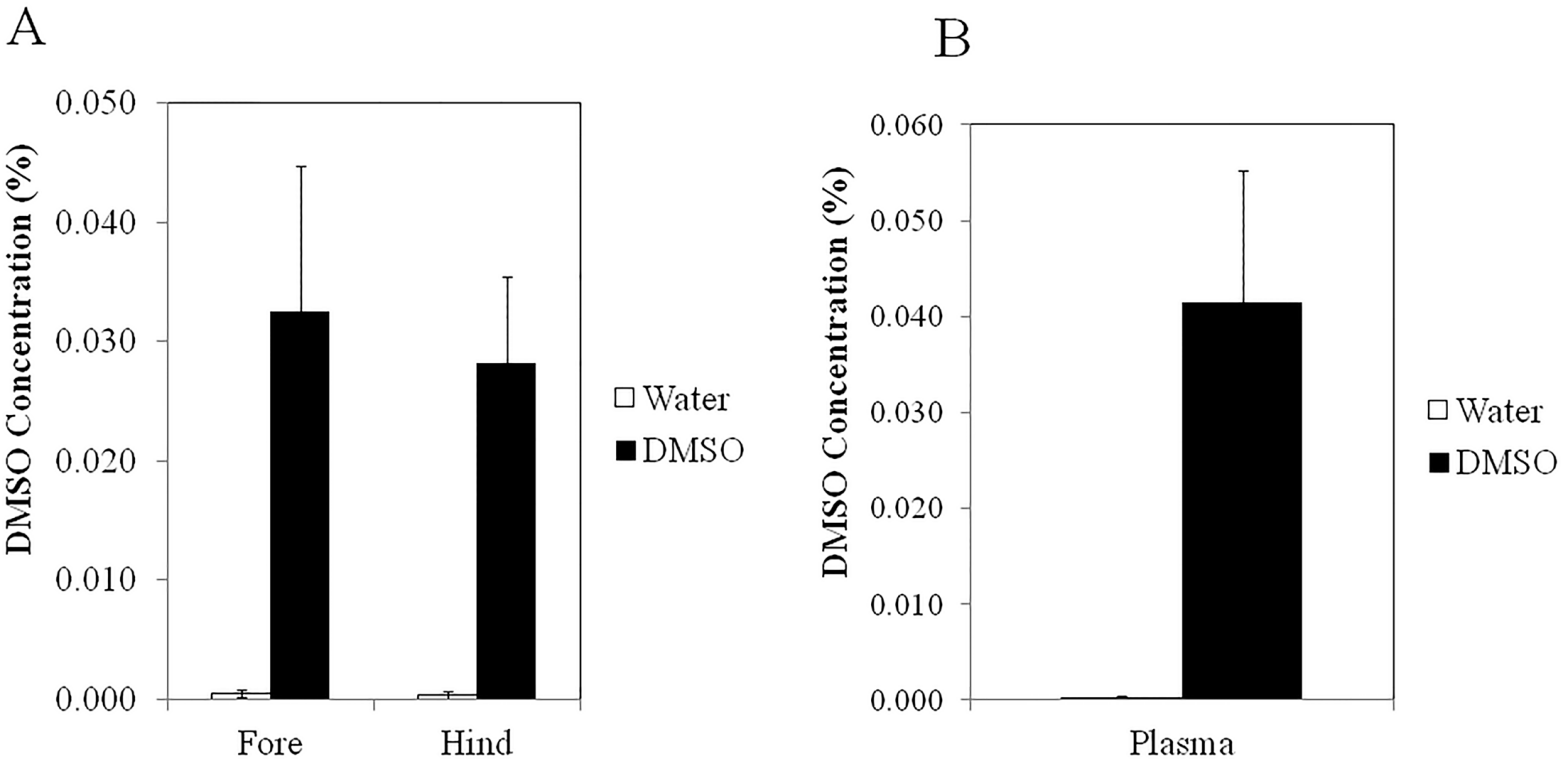
DMSO levels in (A) hind and fore joints and (B) plasma of K/BxN mice. * denotes significant (P<0.05) difference between control (n = 7) and DMSO-treated mice (n = 9).

The ability of DMSO to significantly reduce arthritis-induced swelling in the front, but not in the hind paws corresponded with the efficacy of DMSO to modulate the gene expression of several pro-inflammatory cytokines that have been implicated in arthritic events (Fig 6). For example, gene expression levels of IL-1β, IL-6, TNFα, CXCL1 and CXCL2 in the hind paws of the DMSO-treated mice were not significantly different from the hind paws of water-treated mice while the fore paws of the DMSO-treated mice expressed significantly (P<0.05) lower levels of pro-inflammatory genes (IL-1β, IL-6, CXCL1 and CXCL2) than the fore paws of mice in the control group. DMSO levels in the joints and plasma of the mice were also quantified. Interestingly, even though DMSO was topically administered to only the hind paws, similar DMSO concentrations were detected in both the fore and hind joints as well as in the plasma of the mice (Fig 7). These results suggest that DMSO diffuses systemically throughout the body.

**Fig 8:**
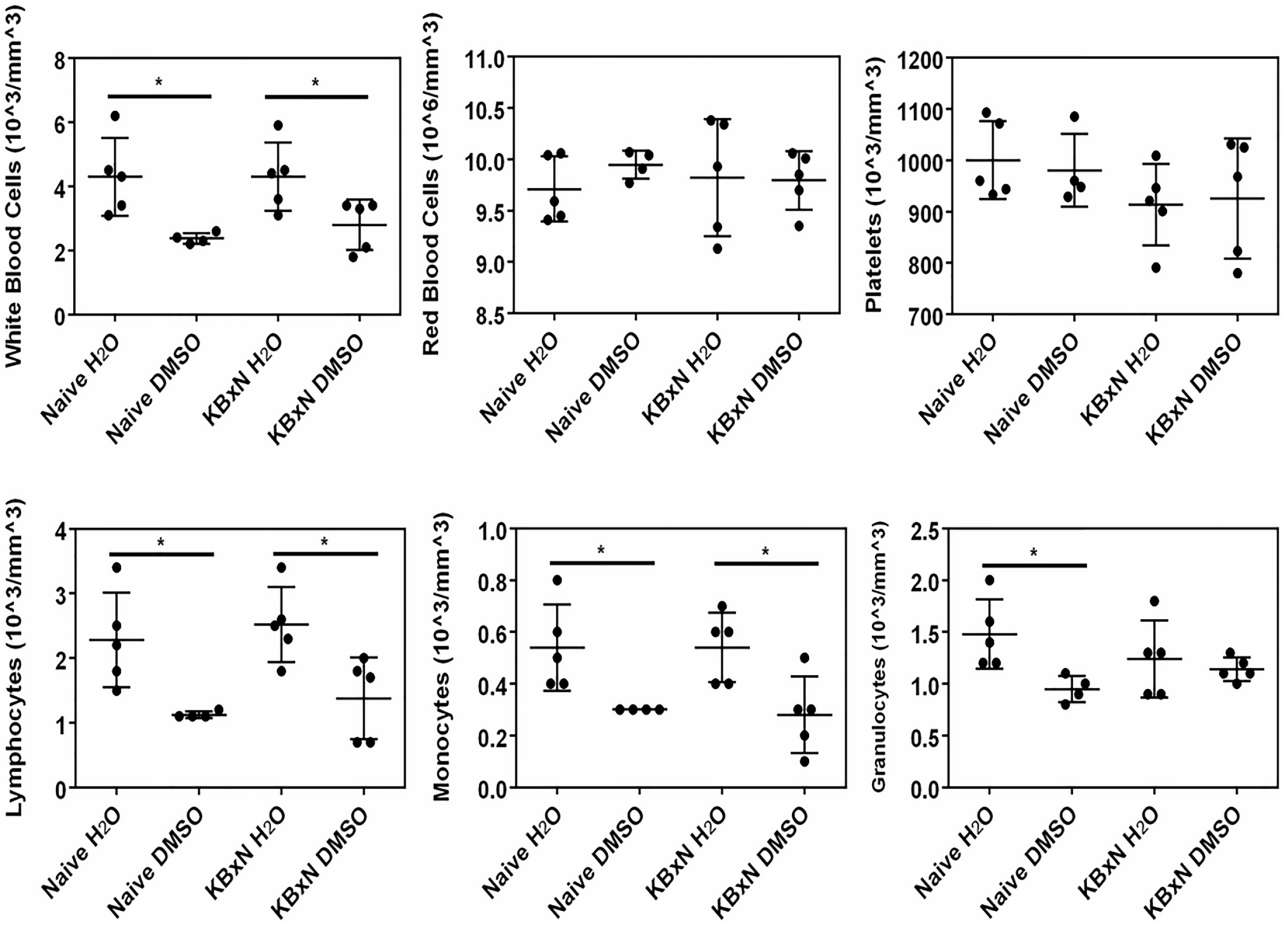
The effect of DMSO in naïve and K/BxN-injected mice on A) blood cell composition and B) white blood cell subtypes. * denotes significant (P<0.05) difference between water and DMSO-treated mice in either naïve, or K/BxN-injected mice (n = 5).

**Fig 9:**
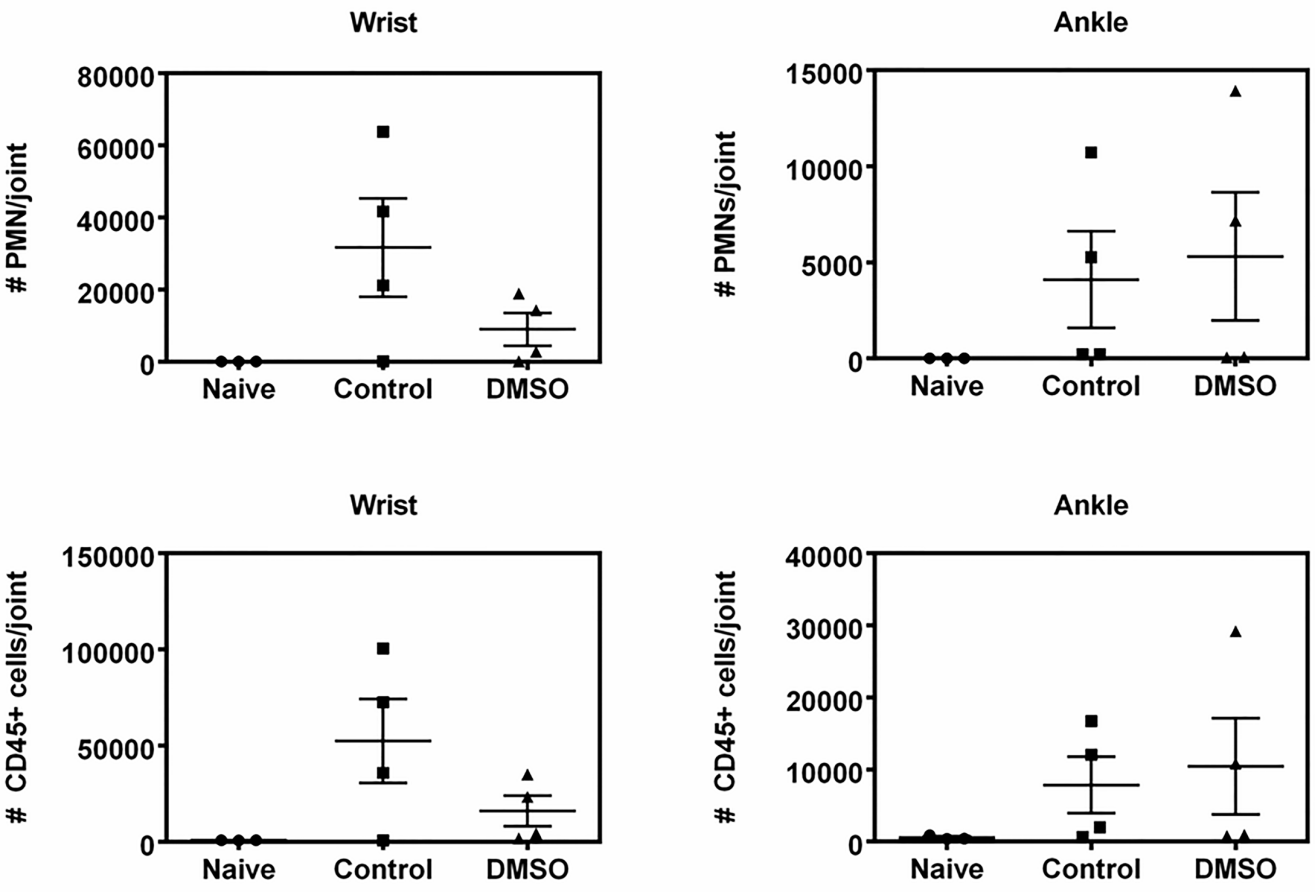
The effect of DMSO in PMN and CD45^+^ cells in the wrist and ankle joints of KBxN-injected mice. Naïve mice serve as control mice not injected with KBxN serum (n = 3–4).

The systemic effect of DMSO was further shown by the significantly (P<0.05) lower wbc numbers, especially monocytes and lymphocytes, in the blood of DMSO-treated mice relative to water-treated mice (Fig 8), regardless of induction of arthritis by K/BxN. A significant decrease in granulocyte content was also observed, albeit only in DMSO-treated naïve mice, and not in K/BxN-injected mice. It should also be noted that the effect of DMSO was specific to wbcs and not to red blood cells or platelets (Fig 8). When the joints were evaluated for the presence of immune cells, it was evident that K/BxN induction promoted recruitment of immune cells to the joints (Fig 9, control versus naïve joints). Interestingly, however, DMSO treatment appeared to reduce the abundance of CD45^+^ cells and PMN cells in the fore but not the hind joints (Fig 9), which corresponds with DMSO being effective in lowering arthritis-induced swelling in the fore, but not hind paws.

## Discussion

We demonstrate herein that DMSO is an anti-inflammatory agent in a whole human blood assay designed to mimic *in vivo* responses to infectious agents. Specifically, we found DMSO potently inhibits the secretion of many *E*. *coli*- and HSV-1-induced cytokines/chemokines at a final concentration of 2%, and some analytes, like G-CSF, IFNγ, IFNα and PGE_2_ are more sensitive, showing substantial reductions at 0.5% DMSO. This anti-inflammatory activity of DMSO is consistent with previous studies reporting a reduction in pro-inflammatory mediators in various model systems [35–37]. On the other hand, we also found that DMSO promotes the release of specific pro-inflammatory cytokines from *E*. *coli* and HSV-1 stimulated blood cells. IL-1β and MIP1α levels, for example, are elevated with DMSO exposure. This finding that DMSO suppresses the expression of some pro-inflammatory cytokines (e.g. IL-8) while promoting others, specifically IL-1β, has been reported previously by DeForge et al [38] using an LPS-stimulated whole human blood assay. This is, however, somewhat controversial since Ahn et al [35] have reported that DMSO inhibits IL-1β production and the efffects of DMSO may be highly dependent on the assay and model systems used. We also found, like DeForge et al [38], that DMSO elicits a biphasic response, promoting IL-6 secretion at low and inhibiting it at high concentrations.

Because DMSO is known to compromise cell permeability and cause cell death at high concentrations [39,40], we investigated whether its anti-inflammatory properties were attributable to its effects on cell viability. Our results suggest that DMSO, up to a 1% final concentration, induces little cytotoxicity to monocytes but a considerable decrease in monocytes is observed at 2%, a mild hemolysis at 5%, and a dramatic decrease in PBMCs at 10%. Thus it appears that DMSO has only a narrow therapeutic window and it is possible the drop in cytokines/chemokines at 2% DMSO is due to lower viability of the monocytes. However, the effects of DMSO at concentrations of 0.5% and lower are likely independent of cytotoxicity.

Despite the finding that DMSO can be cytotoxic above 2% *in vitro*, oral/topical administration of 70–100% DMSO has been reported to result in low systemic toxicity *in vivo* [23,41–43]. One possible explanation for this is that *in vivo*, DMSO is rapidly metabolized to DMSO_2_ and DMS and excreted, primarily through urinary excretion or in expired air, respectively [3]. Because of this we thought it important to look at the immune modulatory effects of these two metabolites and found in our whole blood assay that DMSO is more effective than DMSO_2_ or DMS at reducing *E*. *coli*-induced IL-6 secretion. Taken together with earlier *in vivo* clearance studies in humans, which show that DMSO_2_ and DMS never reach plasma levels approaching DMSO levels [23], it is likely that DMSO, and not its metabolites, is responsible for its reported anti-inflammatory properties *in vivo*.

We next looked at the cell signaling pathways that DMSO might be modulating to affect the production of cytokines/chemokines. For these studies human monocytes were chosen since they were found to be the cells primarily responsible for cytokine/chemokine production in response to an *E*. *coli* challenge. From these studies we conclude that *E*. *coli* triggers the activation of the PI3K/Akt and the three MAPkinase pathways: p38, ERK1/2 and JNK. To see which pathways were critical for IL-6 production we employed pathway specific inhibitors and found that the PI3K, ERK1/2 and p38 signaling pathways are indispensable for the production of IL-6 while inhibition of JNK signaling is not involved. Related to this, we found that DMSO inhibits the ERK1/2, p38, PI3K/Akt and JNK pathways signaling pathways. Considering that DMSO is a solvent capable of disrupting membrane integrity [39], it is conceivable that DMSO is carrying out this inhibition through a non-specific mechanism. However, our findings are also consistent with the previously reported ability of DMSO to act as a hydroxyl radical scavenger (i.e. an antioxidant), since MAPkinase and PI3K/Akt signaling pathways contain redox sensitive sites that can be stress-activated in response to inflammatory stimuli [44,45].

DMSO has been touted as efficacious in the treatment of cancer, in part via its ability to induce the differentiation of some cancer cell lines [46,47]. The recent shut down of a DMSO clinic by the FDA in 2013 after a suspicious patient death only further confirms the need for more *in vivo* work to demonstrate the safety and efficacy of DMSO for cancer treatment [48].

Before embarking on *in vivo* studies in mice we first compared plasma DMSO levels following IP, oral and topical administration at doses that, according to the literature, were the highest without being toxic [49]. As shown in Table 3, highest DMSO levels were achieved via IP administration, with topical giving the second highest. However, given that IP injections would be difficult for people to self-administer and may not give as high local skin concentrations as topical treatments we opted to pursue *in vivo* studies using topical DMSO.

**Table 3:** Plasma DMSO levels in mice following different routes of administration.

	Intraperitoneal^a^	Oral^b^	Topical^c^
**Plasma DMSO (%)**	**0.32–0.76**	**0.004–0.04**	**0.06–0.25**

^a^30 min after IP injection of 4g/kg of DMSO (n = 8)

^b^Oral consumption of 2% DMSO in drinking water (n = 10)

^c^Topical administration of 70% DMSO for 30 sec to hind paws 2 (n = 10) and 4 times (n = 4)

We chose the B16 mouse melanoma cell line for our *in vivo* evaluation of DMSO since this cell line has been shown to differentiate in response to DMSO [31] and *in vivo* tumors can be treated topically. However, even though we indeed found that DMSO promotes the differentiation of these cells, as evidenced by increased melanin production and reduced proliferation, our *in vivo* studies suggest that DMSO, administered at a 70% concentration by topical exposure four times a day to the shaved back of melanoma-implanted mice does not alter the growth rate of tumors when compared to mice treated with water only. An analysis of IL-6 levels in the plasma and the tumors of DMSO- and water-treated mice also revealed no difference, suggesting that DMSO did not alter the systemic inflammatory state of these mice, perhaps because therapeutic levels of DMSO were not reached with this protocol. On the other hand, it is possible that tumor growth was not reduced because some cytokines, like the more DMSO sensitive G-CSF and IFNγ, were inhibited and this reduced the ability of the immune system to restrain tumor growth. Related to this, we looked at the effect of DMSO on macrophage polarization and found that it reduced both M1- and M2- skewing, starting at 0.25% concentration. It is thus possible that DMSO was ineffective *in vivo* because it dampened down the anti-tumor M1 phenotype of the infiltrating macrophages. This is in contrast to a recent study by Deng et al [50], who IP injected DMSO to 4T1-tumor bearing mice and found this suppressed tumor growth by polarizing their tumor associated macrophage from an M2- to an M1-phenotype. It remains to be determined if the difference in our results is attributable to the mode of administration or the tumor cells utilized.

DMSO is currently widely used in inflammatory conditions such as rheumatoid arthritis and clinical trials are underway, although it is difficult to derive any conclusions from these trials since the garlicky odor generated from DMSO’s metabolite, DMS, prevents a truly double blind study [16,51]. In our studies, arthritis was induced by IP injection of K/BxN serum to C57BL/6 mice. Topical exposure of the hind paws to 70% DMSO, administered twice daily, showed a trend towards a reduction in the swelling of the hind paws and a more marked, statistically significant reduction in the swelling of the front paws. This was associated with a lower gene expression of a number of pro-inflammatory cytokines and chemokines, compared to water-treated mice. This is consistent with what we observed in the blood assay where DMSO reduced the expression of pro-inflammatory cytokines, with the exception of IL-1β. However, there are many possible explanations for the difference seen between the slightly elevated IL-1β mature protein levels in the 7 h whole human blood assay after DMSO treatment and the lower *in vivo* IL-1β mRNA levels found in the front paw joints after 7 days of DMSO treatment, the most likely being that DMSO acts to reduce neutrophil recruitment to the synovial joints, which leads to a reduced presence of pro-inflammatory cytokines such as IL-1β. Neutrophils are thought to play an essential role in the initiation and progression of arthritis in this K/BxN model system and were shown to be the main type of immune cell present in the synovial joint during inflammation [52–54]. The finding that DMSO lowers wbc numbers in blood and reduces mRNA levels of IL-6, CXCL2, TNFα, IL-1β and CXCL1 in the fore paws of DMSO treated mice, coupled with the observation that fewer CD45^+^ cells (total leukocytes) and PMN are observed in the fore joints, but not the hind joints of DMSO-treated mice, supports our hypothesis that topical administration of DMSO systemically suppresses wbc levels, perhaps via DMSO’s effects on G-CSF levels, that in turn reduce recruitment of neutrophils to the joints and mitigates arthritic events from occurring. To test this hypothesis we exposed mouse bone marrow-derived mФs to various DMSO concentrations for 3 days (to mimic *in vivo* exposure times) and found that while these DMSO concentrations did not reduce the viability of these cells (S1 Fig), concentrations as low as 0.3% (a concentration comparable to that found in mouse plasma after topical administration of DMSO) significantly inhibited LPS-induced G-CSF production (S2 Fig). Importantly, our finding that fewer CD45+ cells are observed in the fore joints of DMSO-treated mice is consistent with a previous report showing that DMSO inhibits the infiltration of granulocytes and monocytes into infected pleural spaces in rabbits [55]. The lower efficacy in the hind joints, despite a similar DMSO concentration in the hind and fore paws, as well as in the plasma, might be explained by the confounding skin irritation that is known to occur with topical application of 70% and higher concentrations of DMSO [56].

To date, studies into the efficacy of DMSO in animal models of rheumatoid arthritis have produced conflicting results. Colucci et al [57], for example, showed that oral administration of DMSO to mice was effective in reducing zymosan-induced edema in the mouse paw, while local administration of DMSO enhanced paw swelling instead. On the other hand, Görög and Kovács (1968) demonstrated in adjuvant arthritic rats, that topical application of DMSO produced a more pronounced anti-inflammatory effect than when given orally [58]. Confounding factors in these studies that could contribute to the discrepancies observed are the dose and route of administration of DMSO as well as the animal models used to recapitulate arthritic events. Topical administration of DMSO in inflammatory agent(s)-induced arthritic rats for example, was thought to increase the penetration of the inflammatory agents into tissues, resulting in a lack of efficacy or an exacerbation of paw edema [59]. However, the etiology of inflammation in the K/BxN mice does not rely on the introduction of exogenous inflammatory agents, but on the recognition of endogenous auto-antigen that triggers the activation of the innate immune system, which more closely resembles the pathogenesis of rheumatoid arthritis in humans.

In conclusion, DMSO is an anti-inflammatory agent that demonstrates efficacy in whole human blood by inhibiting the ERK1/2, p38, JNK and PI3K/Akt signaling pathways in human monocytes during inflammation. This is associated with a reduction in the production of inflammatory mediators such as G-CSF at low DMSO concentrations. This in turn likely leads to a systemic suppression of wbc levels that contributes to DMSO’s efficacy in reducing arthritic events in the K/BxN mouse model and its inability to slow B16 melanoma growth. We therefore conclude that the use of DMSO as an anti-inflammatory agent in conditions such as rheumatoid arthritis may have some merit but cannot support its use as an anti-cancer agent.

## Supporting Information

S1 Fig DMSO does not affect the viability of bone marrow-derived mФs cultured in M-CSF and stimulated with LPS (10 ng/mL) for 3 days as determined using the MTT assay. Results are expressed as a % of non-DMSO-treated cell viability (n = 3).
(TIF)

S2 Fig DMSO reduces G-CSF production from bone marrow-derived mФs cultured in M-CSF and stimulated with LPS at 10 ng/mL for 3 days. G-CSF levels were determined by ELISA (R&D Systems, Minneapolis, MN) according to the manufacturer’s instructions. * denotes significant (P <0.05) difference relative to non-DMSO-treated cells (n = 3).
(TIF)

## Abbreviations

Arg-1: arginase 1
DMS: dimethyl sulfide
DMSO: dimethyl sulfoxide
DMSO_2_: dimethylsulfone
*E. coli*: *Escherichia coli*
GPI: glucose-6-phosphate isomerase
HSV-1: herpes simplex virus-1
IL-1β: interleukin-1β
iNOS: inducible nitric oxide synthase
IP: intraperitoneal
LPS: lipopolysaccharide
mФs: macrophages
MIP-1α: mФ inflammatory protein
MTG: monothioglycerol
NO: nitric oxide
PBMCs: peripheral blood mononuclear cells
PGE_2_: prostaglandin E_2_
TLR: toll like receptor
wbc: white blood cell
