# Supplementary figures and images for: DMSO Represses Inflammatory Cytokine Production from Human Blood Cells and Reduces Autoimmune Arthritis
Source: PLoS One. 2016 Mar 31;11(3):e0152538. doi: 10.1371/journal.pone.0152538 (PMC4816398; doi:10.1371/journal.pone.0152538)

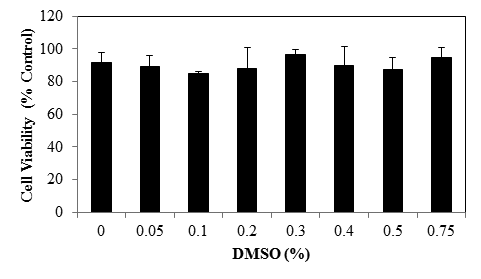

Supplement: S1 Fig — Results are expressed as a % of non-DMSO-treated cell viability (n = 3). (TIF) [file pone.0152538.s001.tif]

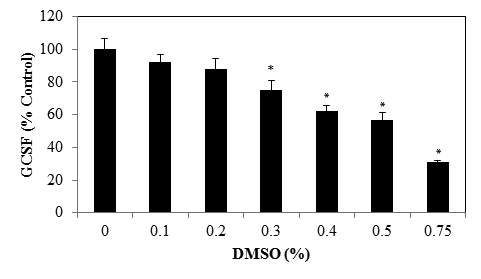

Supplement: S2 Fig — G-CSF levels were determined by ELISA (R&D Systems, Minneapolis, MN) according to the manufacturer’s instructions. * denotes significant (P <0.05) difference relative to non-DMSO-treated cells (n = 3). (TIF) [file pone.0152538.s002.tif]
